# Reintegration Among High-Profile Ex-Offenders

**DOI:** 10.1007/s40865-018-0093-x

**Published:** 2018-09-29

**Authors:** Marieke Liem, Daan Weggemans

**Affiliations:** Institute of Security and Global Affairs (ISGA), Leiden University, Turfmarkt 99, 2511 DP The Hague, The Netherlands

**Keywords:** High-profile, Media, Public attention, Reentry, Desistance, Life course theory

## Abstract

**Purpose:**

The reintegration of high-profile ex-offenders—including homicide offenders, pedophiles, and terrorists—frequently receives great political and public attention. This raises several important questions: how do such offenders reintegrate into society after their release? What is the impact of their prison sentence and media attention on life domains post-release? And, given their presence in the public eye, how do current life course theories account for desistance among this special group?

**Methods:**

Based on in-depth life course interviews with ten Dutch high-profile ex-offenders and interviews with 17 professionals involved in their reintegration, this study seeks to address a significant gap in academic literature on the role of public attention on reentry and desistance.

**Results:**

While none of the subjects reengaged in criminal behavior, all of them significantly struggled in the domains of family relations, parenthood, intimate partner relationships, employment, and housing post-release. This poses challenges in terms of explaining their desistance through life course theory alone. They are frequently in the public eye, which—combined with strict supervision—inhibits them from rebuilding relationships that may act as informal social controls.

**Conclusions:**

Findings emphasize the significance of the broader social context of high-profile offenders as well as factors such as time and age, for gaining an understanding of their lived experiences and desistance.

## Introduction

The societal reentry of some ex-offenders receives a great deal of political and public attention. Well known are the public demonstrations and expressions of anger and concern against released sex offenders [5, 12, 51]. But also, the release of other individuals—including convicted homicide offenders and terrorists—may generate large-scale societal disturbance. While such sentiments may be understandable from a moral perspective, it raises several important questions: how do such offenders reintegrate into society after their release? How do they desist and what role does the vast amount of media attention play in this process? Homicide offenders (e.g., [2, 28]), terrorists (e.g., [45, 48]), and sex offenders (e.g., [5]) are separately each subject to scholarly investigation, yet are seldom studied as a group. Notwithstanding their varying criminal backgrounds, these delinquents may face similar experiences post-release. One example includes so-called high-profile ex-offenders, who share at least three particular characteristics. First of all, those who have been convicted of sex offenses (especially when children are victimized), acts of terrorism, and homicide each undermine a community’s perception of public safety [27, 28, 53]. Second, following their offense, all three types of offenders are typically incarcerated for extended periods of time, not infrequently, due to their offenses, in separate prison wings away from the general prison population [15, 53]. After years of confinement, the consequences of long-term imprisonment come to the forefront. Those sentenced to long-term incarceration are more likely to lose pro-social contacts in the community and become removed from legitimate opportunities such as work and education after release [19, 28, 30, 31]. Finally, the reentry of these offenders brings about a new wave of societal unrest, particularly related to the fear that “they will do it again.” The question that remains, then, is how successful transition from prison to society can be ensured?

This article seeks to discuss the impact of imprisonment and public attention on different life domains after release. Over the last decades, the life course approach has offered an important perspective for scholars seeking to understand processes of reintegration and desistance post-release [20, 43]. According to this perspective, the likelihood to desist increases as so-called stakes in conformity increase, such as ex-offenders taking up roles as an intimate partner, parent or employee [47, 51, 52]. However, the bulk of life course research has focused on the general ex-offender population (e.g., [14, 21, 51]). While not short of attention in general, high-profile offenders and the effects of large-scale public attention on reintegration in general, and desistance in particular, have readily been neglected in this realm of scientific inquiry. It remains to be questioned if and to what extent such high-profile are able to successfully reenter, given the condition that they are frequently in the public eye, combined with strict supervision.

## Life Course Theories on Desistance

From a life course perspective, stakes of conformity associated with intimate partner relationships, parenthood, and preexisting family ties are all thought to contribute to successful reentry [29]. A life course theory point of view, in short, posits that conventional pillars of social control, such as family relations, parenthood, and intimate partner relations, are key to understanding desistance [21]. Following Sampson and Laub [43], criminal behavior changes as important life events change—the stronger the ties to family and work, the less the criminal behavior. In this view, the positive influence of a spouse or an employer, as well as assuming a role as a parent, creates a social dynamic that produces informal social control. Prior research has shown that assuming traditional roles within a family, such as a parent or a spouse, can benefit the reentry transition process as it aids in the development of pro-social identities [47, 51, 52]. Living with a spouse may give one “more to lose” or “increase shame” in committing crime [18]. In addition, living with an intimate partner may significantly influence the nature of daily activities, suggesting that these lifestyle changes may also work to limit involvement in criminal behavior [14]. It is important to note that marriage “by itself” and employment by itself does not support desistance; rather, it is the strength and quality of marriage (cohesive relationship) and employment (job stability, commitment to work, mutual ties binding employees and employers) that predict desistance [25, 28]. In addition to considering newly assumed social roles as a parent or intimate partner, research has also focused on the role of preexisting family relations in providing informal social control. José Cid and Joel Martí, for example, found that preexisting social bonds, such as family ties, were fundamental in moving away from criminal behavior. In this dynamic, the offender ceases criminal behavior in order to compensate for the supportive role of the preexisting relationship [8]. Further, the exercise of informal social control is not only limited to the nuclear family but also includes larger social networks and institutions, such as religious and volunteer groups [9]. Social support can thus stem from a variety of sources that have been shown to contribute to desistance. Together, these “webs of conformity” provide not only constraints but also supports [28].

In this article, we mostly focus on primary desistance [11] or “act-desistance,” referring to a periods of non-offending. However, at this point, it is important to emphasize that desistance is a complex process, which does not only consist of act-desistance (non-offending) but also includes “identity desistance” (for the internalization of a non-offending identity) and “relational desistance” (for the internalization of change by others) [37]. In this context, it is also claimed that successful desistance often depends on the presence of others, who, as Herman puts it, “redefine the person in a non-deviant light” ([17]: 564). Indeed, the instigation of a trajectory of desistance depends on the individual’s decision to leave crime. However, the final aspect of “going straight” is to “become normal” in the eyes of others, which allows the individual to fully achieve a social identity as noncriminal. Meisenhelder links this process of “delabeling” as the “certification” phase of desistance—“the social verification of the individual’s reform” ([36]: 137). In certification, he elaborates, “some recognized members of the conventional community publicly announce and verify that the ex-felon need no longer be considered a criminal but rather should now be treated as a normal member of the social group” ([36]: 139). Meisenhelder’s argument is that for successful desistance, it is not only necessary that an individual accepts conventional society, but that conventional society also must accept—preferably through a set of formalized communicative actions—the individual, which is also present in the work of, for instance, Maruna and colleagues [35]. They argue that “ex-offenders need to be morally and socially reintegrated, but they also have to feel that this reintegration has been justified by their own efforts to “make good” and redress past crimes” ([35]: 279). Maruna et al. conclude that “without some concrete recognition of their reform (i.e. some “certification”), many ex-offenders might not be able to maintain the difficult process of “recovery” and desistance” (ibid). It remains to be questioned to what extent high-profile ex-offenders—given the nature of their crimes and media interest—ever receive such certification.

In sum, marriage, parenthood, employment, and preexisting family ties are thus thought to constitute mechanisms of informal social control that facilitate the alteration of criminal trajectories, putting ex-offenders on a path toward desistance [18, 21, 50]. Successful participation in a personal relationship, a job, or some other conventional area of life thus provides personal rewards and reinforces a noncriminal identity [47]. Even though this notion has been widely tested cross-culturally, among various offender groups [4], so far it is not clear whether this theoretical approach effectively captures the experiences of those who have been in the public eye ever since their arrest, conviction, and release. One may question to what extent these offenders are able to engage in pro-social relationships post-release, given the nature of their offense, subsequent public attention, and associated stigma. Particularly, the latter notion is of importance when dealing with this special group: rather than formal restrictions to certain types of employment, the stigma of being an ex-offender may inhibit ex-offenders from establishing social relationships. Previous studies have outlined three ways of managing the stigma of being an ex-offender. These include hiding the fact that one has been in prison (secrecy), avoiding social interaction (withdrawal) and education (preventative telling) [23, 54]. In deciding if, and when, to disclose, ex-prisoners must weigh the costs and benefits of disclosing. The benefits of disclosing include not having to worry about hiding it, finding others who can help or express approval, and promoting a sense of personal power. In contrast, the costs of disclosing include being excluded from opportunities such as employment and worrying about what people are thinking about you [23]. For high-profile ex-offenders, one may argue that the only option available for them is withdrawal, as simply revealing their name is enough for others to know who they are.

## Researching High-Profile Offenders’ Lived Experiences

In order to empirically ground our argument, we now turn to the experiences of high-profile offenders and professionals in the Netherlands. One of the key organizations involved in the post-release phase is the Dutch Probation Service. Over the last decades, probation has become a central element in the criminal justice systems of many Western countries [39]. Among other things, Probation Service in the Netherlands is responsible for monitoring an offender’s conditional release. The conditions imposed on probationers may, for example, include periodic reporting to the probation officer, wearing an ankle bracelet, and area and contact restraints. The Dutch Probation Service is also formally tasked with providing some practical support to criminal offenders and suspects (for instance through providing assistance in access to education, employment, and behavioral therapy). Each municipality, in turn, is responsible for providing support in the domains of housing, work and income, debts, and health care.

### Sample

To determine who could be regarded a “high-profile offender,” the media database LexisNexis was used, to search in Dutch local, regional, and national newspapers and websites with keywords specific to the research population.^1^ With the aim of gaining an insight into relatively recent lived experiences, we limited our search to articles that were published between 2005 and 2016. This has resulted in a total of 4462 media reports.

Localizing these high-profile (ex-offenders) was a first step, gaining contact with them a second. Many former prisoners are subject to feelings of shame, mistrust, and some want to leave the past behind [33, 34]. Previous experiences of stigmatization also often function as barrier for participating in (scientific) research [42]. In 29 media reports, a reference was made to the respective lawyers of the offenders found in the media reports. We requested each of these lawyers whether their clients would be willing to participate in this study. Recruitment through gatekeepers is a common method for recruiting subjects that are difficult to approach [3]. Some lawyers told us that their high-profile clients were, indeed, unwilling to cooperate due to feelings of mistrust, shame and fear. In two other cases, contact with research subjects mentioned in the media reports was established directly via e-mail. Four other interviews were arranged by means of the networks of the researchers.

These sampling methods resulted in interviews—conducted between November 2015 and June 2016—with ten high-profile former prisoners. These respondents have been sentenced for one or more sexual offenses (*N* = 5), homicide offenses (*N* = 2), or offenses committed with a terrorist or extremist intention (*N* = 3). All the respondents were male and aged between 25 and 65 years when released from prison. The former prisoners with a terrorist background were on average the youngest group and those sentenced for sexual offenses the oldest (see Table 1 below). They were incarcerated in the last two decades and, at the moment of the interview, were released between 1 and 9 years ago.

**Table 1:** Basic characteristics respondents at the time of interview

Respondent	Criminal offense
Johan	Sexual offense
Otto	Sexual offense
Willem	Sexual offense
Greg	Sexual offense
Hank	Sexual offense
Marco	Homicide offense
Peter	Homicide offense
Youssef	Terrorist offense
Ahmed	Terrorist offense
Khalil	Terrorist offense

The interviews were semi-structured. This means that a number of topics were determined from which the interviewers could deviate when deemed relevant. These topics included elements from life course theory (such as romantic and social relations, parenthood, and employment). This interviewing method offers as an advantage the opportunity to ask respondents for additional questions and details, in order to gain a more in-depth understanding of their experiences [16]. The interviews had a time frame of 1.5 to 4 h and were conducted in a familiar environment (e.g., a respondent’s home, workplace, or at the house of a friend). Three interviews were held in lunchrooms and one interview took place in our University office.

In addition, we interviewed 17 professionals working with high-profile offenders. They are all regarded as specialists on the issue and work at the Ministry of Security and Justice, Dutch Probation Services, Public Prosecutors office, a penitentiary, NGOs, municipal level, or in a penitentiary. With these semi-structured interviews, we sought to map the general practice of reintegrating of high-profile offenders. We asked the professionals about the influence of public and political attention on these offenders, as well as the effects of such attention on the practice of reintegration. These interviews offered us the opportunity to cross-check some of the claims made by the high-profile offenders. Most interviews were conducted in a face-to-face setting at the professional’s office. In one case, the interview was held over the telephone.

With the interviewees’ permission, most interviews were audio recorded. Interviews were transcribed ad verbatim. The interviews were submitted to content analysis. Following prior research in this area [24, 28, 41, 46, 53], data was coded using predetermined themes based on the topic list as well as emergent themes. The thematic coding framework included perceptions of stigmatization, role of media and politics, social and intimate relationships, family ties, experiences with finding work, housing, and income and support during reentry. Three researchers were involved in identifying the coding categories. Subsequently, the framework was used for assessing all interview transcripts. Data was coded by one analyst and verified by another analyst. The analysis looked for patterns within the themes, focusing on the impact of the prison sentence and public attention on the different life domains. Below, the emerging issues and themes are characterized by participants’ quotes.

### Ethical Considerations

Given the well-known identity of the interview participants, numerous precautions were taken to secure anonymity and confidentiality. First, interviews were held at locations suggested by the interviewees, where they indicated they felt safe to talk to us—ranging from their private home to public venues. We informed all respondents about the research design and goals via e-mail prior to the interview. At the interview, verbal consent was provided in all cases. In order to guarantee the safety and privacy of the respondents, all personal information from the interviews has been made anonymous in the transcription process. Even through some interviewees wanted their own name to be used, we replaced each name with a pseudonym. To avoid identification, we also left out or changed specific information about their persona—including hobbies, place of residence, or previously held jobs. No identifying information was retained following the completion of the analysis. Finally, we e-mailed each participant a word file including interview excerpts we intended to use, for their review, and only included these excepts when they approved.

## After Release

None of the interviewed offenders self-reportedly reoffended post-release. At the same time, the high-profile nature of their offenses inhibited the building of conventional social relationships through family, intimate relationships, or children and employment that may act as potential deterrents for future crime. What we should therefore be questioning is not only how they accomplished their desistance but also to what extent desistance corresponded to being successful in each of the aforementioned life course domains. Below we discuss both the offenders’ lived experiences as well as the professional reflections on developments taking place within these domains.

### Intimate Relationships

Nine out of the ten interviewed ex-offenders were involved in a romantic relationship prior to their incarceration. In four cases, the relationship ended while they were in prison, which was both attributable to their prison sentence, as well as to the (public) nature of their crime, as Greg, convicted for a sex offense with a minor, points out:

“You are portrayed as the most dangerous criminal. One that could strike any minute. [...] My family was predominantly informed by the media rather than by experts. These experts told them that they are ‘not responsible for who will show up on their doorstep’. They are not involved in any way, while they should be. My ex-wife believed the words of the public prosecutor rather than those of me and my lawyer.”

In five cases, intimate relationships survived the (at times prolonged) period of incarceration. Post-release, interviewees pointed out that the quality of their relationships took a big hit, not only because of their time away in prison but also because their intimate partners were faced with the effects of their crime. Family relations became an issue of concern for them. For example, Peter talks about his wife’s frequent reproaches:

“My daughter has always been very open, also from the moment I was arrested [...] but my son has always hidden it from his friends. My wife still frequently argues that my children have not accomplished what they could have when “all of this” did not happen.”

Willem, who is now in his 60s, similarly reflects on the struggles in the relationship with his partner:

“I still have my partner after 30 years. [...] Yet, it has been a very difficult period and I worried how our relationship would survive it [prison]. He [partner] had a very difficult time [...]. We lost many mutual friends […] [and the] media attention for my case was confronting for him”.

These quotes illustrate that, due to the public attention for their crimes, their intimate partners not only felt to be in the public eye but also experienced that their social environment approached them suspiciously, almost as if they were co-offenders in the same crime(s).

Those not involved in a relationship prior to incarceration, but who wanted to get involved with someone, also struggled, predominantly, with stigma management. Some said that stigma prohibited them from having intimate relationships and adopted a withdrawing approach to stigma management. Others, like Marco, adopted secrecy, as he argued “[I] don’t see the point of telling them [other people].” When they do open up to new relationships, they always do so with caution and with low expectations, also known as preventative telling, as Greg’s example illustrates:

“Well, I always wait-and-see, trying to find out how someone would respond to things like these. […] Eventually I will tell everything, about who I am and what I am convicted for. I told her to take a few weeks to process everything, to think things through. ‘Have a couple of nights sleep and then let me know if I would still be welcome or not. I do not expect anything from you, and I will not be angry if you decide I’m not’. I told her I would understand”.

### Parenthood

In total, five ex-offenders were fathers prior to incarceration, two of whose children had already left the house. The other three had children who were minors at the time of their incarceration. One example is Peter, a man in his 60s, who was arrested when his children were still young. During his 12-year incarceration for homicide, his wife was “pretty much on her own” in taking care of the children.

By the time they were released, their children entered adolescence or even adulthood. In Youssef’s case, for example, his ex-wife did not want to be associated with his case and broke off contact after his arrest. He resigned to his ex’s decision and due to the fact she lives abroad, Youssef—now in his late 20s—has had no role in raising his daughter since. Rather, due to the nature and duration of their incarceration, these men did not take up a significant parental role while in prison. Further, the length of their sentences disrupted their life courses. Once they were released, they were “out of time” in fulfilling a parental role that could have acted as a deterrent. Others considered themselves as too old to start a family, as Marco, who is now in his mid-40s, illustrates:

“My life is rather stable at the moment [...] but still my life will always be different than of other people. I mean, […] when I see people of my age; they have bought a house, have a family. Well.. I will never have that.

Parenthood, in other words, lacked the potential to lead these men to paths of desistance. Ahmed—who is in his late 20s—seems to be an exception to this rule, as he considers his identity and role as a father as very important and, as such, possibly functions as a deterrent.

### Preexisting Family and Social Ties

With the exception of two, the vast majority of the interviewees said they lost contact with family members, as well as with close friends, while in prison. They partly attributed this to the length of the confinement, as well as to the nature of their crime(s) and media coverage of their stories. Greg, for example, who is now in his 60s, is only in touch with one of his sisters. While in prison, his daughter, among others, broke contact with him:

“My son-in-law has, among other things, pressured her to do so: ‘If you keep seeing your father, then I will leave you!’ he’d said”

Youssef, who is still in contact with most family members, lost contact with his brother post-release:

“I rarely speak to my brother anymore. I do not see him very often. [...] He also experienced quite some problems because of the negative label I have been given.”

In line with previous research [53], at this point, it must be remarked that while all interviewees with a terrorist background experienced substantial negative consequences of the high-profile nature of their cases, most maintained contact with their relatives. Both Youssef and Ahmed also managed to make new friends; however, in both cases, these friendships were formed while incarcerated or on the basis of their high-profile status and could therefore hardly be perceived as strong, pro-social ties.

Most other respondents speak of not only of a lack of contact with family members but also to a more general lack of social contact, in some cases resulting in far-reaching social isolation, as Hank illustrates:

“There is absolutely nobody who wants to be associated with me. [...] I am always alone, well practically always. I do not really know anymore what loneliness is, since it is my life. I only have contact with a couple of people. Most of them of online. That is the safest way for me. Because the more people you bring together, the bigger the odds that people will recognize me. […] Those who I speak with recognize my problems and we are able to talk about them.”

At the same time, interviewees emphasize that a supportive social network is essential for effective reintegration. Interviewed professionals even claim that this particularly accounts for high-profile offenders “especially when people are under a magnifying glass and there is social unrest, then it is important that they are supported by friends or family members”; one professional involved in their reentry puts it. However, they readily acknowledge that building and maintaining such a supportive social network is problematic for this group of ex-offenders:

“Indeed, while being one of the keys to success, it is very difficult to build a healthy social network through work or organizations. But who wants to befriend these notorious offenders? Only a really small group of people. In practice, volunteers are often the only ones willing to give them a chance [...] in terms of being supportive factors. […] I think that is absolutely crucial given the fact that society has already written them off and flushed them down the toilet.”

Difficulties in reentering in the spotlights also become apparent when it comes to their social networks. Six of the ex-offenders told us that they have made some new friends in prison, while most of them have been unable to meet new people after their release. Both Marco and Ahmed, for example, concluded that most of their friends are still in prison or have recently been released.

### Employment

Post-release, the vast majority of those interviewed struggled with finding employment—not only because their (extended) incarceration, which inhibited them from applying for certain jobs, and public profile, which seemingly inhibited them from being hired, but also due to the lack of assistance of formal reentry organizations, as Johan explains:

“I had hoped that the Dutch Probation Service and municipality would help me in finding a job. Yet, that didn’t happen really. I did receive valuable assistance by a volunteer organization. They visited me and helped me making a CV. However, in the end no-one actually found me a job”.

Even though one may question Johan’s sense of agency in individually acquiring a job, the probation service is certainly required to play a supportive role in obtaining employment. Professionals at various reentry organizations (including the probation service), however, supported these ex-prisoner’s accounts of experiencing a “hands-off” approach, given the public sensitivity of these ex-offenders. Youssef, who was convicted for a terrorist offense, similarly experienced a lack of support, seemingly due to the nature of his past offense and public profile, as he exclaims:

“Help me then! I am being punished twice here. Someone from Dutch social services told me: ‘How do I need to find a job for you? How would an employer respond when I say ‘hey, this is Youssef, a well-known former terrorist?’ He told me that in the presence of my wife!”

These practices, as Youssef and others outline, seem to be indicative of a situation in which professionals do not know how to effectively assist high-profile offenders. Moreover, in some cases, professionals even seem to act out of risk management, rather than reentry support, or as Youssef puts it elsewhere: “they seem to think ‘imagine when things go wrong here’.” Professionals’ accounts support this sentiment, as one public prosecutor says: “While we know from experience that assisting in the reintegration of offenders can be beneficial, at some point things can turn into a political discussion [about perceived risks].” This seems to be especially the case when media enter the equation or as one policy officer for local governments states: “the less spotlights, the better. When the cameras come in, the potential actions of governments are limited”.

Ultimately, some ended up being unemployed, while others took up a voluntary work. While this provided them with some satisfaction, they are also constantly aware of possible restrictions to their activities, as Johan exemplifies:

“I am volunteer at a church, I work in the garden. I also like to be involved in providing technical services there and I organize an open museum day. Well, that is be something the Probation Service would probably forbid when they would find out. Because of the presence of children there”.

For some, volunteering became a way to find meaning in their lives, yet at the time of the interview, none of the interviewees mentioned volunteering as a part of their identity. Difficulties in finding employment are readily acknowledged by many professionals. In line with ex-offenders, they too sometimes wonder “who is going to employ this particular offender?”

### Housing

Oftentimes offenders were unable to return to their old homes. For example, when we asked Greg about his release from prison, he admitted that finding a house had been rather problematic: “I was forced to live on the streets for a while, because Probation Services could not find a house that was suitable for someone like me.” Ultimately, he states, “The judge and public prosecutor thought I was too big of a risk when living on the streets. Eventually, I had to finish the remaining three months of my sentence behind bars.”

In total, more than half of the former prisoners we interviewed experienced problems in finding housing. Support from official governmental organizations was experienced as insufficient. In some cases, it took months—after their release—before a location was found, in others they were unable to be released in time because of a lack of housing, and in other cases, the only available housing was far removed from their original place of residence. Ahmed, for example, was not allowed to return to his old home because of the conditions of his release (he received an area ban) and, as such, he ended up in a house and city in which he did not want to live. His experience is similar to Khalil’s:

“I did not want to live in this city, because I [whilst incarcerated] already knew I would go to a school somewhere else; my future would not be there. I thought “What on earth should I do in this other city?”

This dynamic was reported by many interviewees, who felt frequent media attention covering their release inhibited their efforts in finding a suitable place to live. Hank, for example, argued that after his release he wanted to return to his own home. However, as his personal safety could not be guaranteed (demonstrators threatened to harm him), he was unable to do so:

“Of course, it [his release] has been announced very broadly that I could not return to my own home […] When journalists write that you have been released from prison, then, yes, people sometimes get the spontaneous idea to pay someone a visit. Not with the best intentions though.”

Hank further states that as the Dutch Probation Service, the public prosecutor and local governments were unwilling to offer him help in finding housing, he went looking for alternatives: “I started living on campsites” he explained. However, sometimes, people recognized him and he was chased away from the perimeter:

“Eventually, an unofficial organization offered to help me. They called the mayor of the municipality in which I lived. Instantly, police cars arrived at the campsite where I lived. They had given me an area ban. I had to leave again […]. Housing-organizations keep refusing me. Even the homeless shelter told me I wasn’t welcome.”

It is worth noting, however, that the degree of stigma in this context may differ between offenders, given their index crime. As one professional emphasized: “When a convicted murderer comes to live next door, well, people probably do not like that. But it is a whole lot less problematic than when someone has a history as a sex offender.” Notwithstanding, these differences in public stigmatization, the Public Prosecutors office and other professionals admit that finding these high-profile ex-offenders a place to live often takes considerable effort. They see a paradox, while emphasizing the importance of reintegrative activities; they also describe the experienced societal and political pressure among professionals and governments. A representative of the Dutch organization of local governments concludes that this is the “five percent for whom no protocol is available, for whom no system can be designed.” The societal pressure is, among other things, fuelled by a fear of what will happen in the case a high-profile offender relapsing into criminal behavior. This makes housing a high-profile offender a potentially unpopular thing to do, as a Mayor’s advisor highlights: “sometimes a Mayor rejects a high-profile offender’s request for housing in his town. (…) For this political support is needed, it is *precious political capital*.”

Reentry of high-profile offenders often generates substantial media attention. This, in turn, leads professional organizations to focus on repression and risk-management instead of finding options for adequate support and, hence, at least in theory, a higher risk of recidivism. Various professionals argue that significant assistance in the transition from prison to society can only be ensured “when someone is a bit invisible” (professional working at reentry organization). This may be achieved by changing their names or physical characteristics. This need for anonymity can also be linked to the lack of public rehabilitation—or in Meisenhelder’s terms, certification—of high-profile offenders. Seldom, infamous sex offenders, murderers, and terrorists are *delabelled* by officials who publicly acclaim that the offender is now an *ex* and no longer a threat. However, as a policy advisor highlights, “the public comforting of experts or relevant officials promotes the reintegration of an ex-offender.” A valuable alternative, according to both professionals and high-profile offenders, is the involvement of informal social networks of volunteers. On a smaller scale, they can redefine the social status of a former prisoner. As one case-worker puts it:

“The public shaming of known offenders is counter-productive. ‘Oh, society has written me off?, I will show them then’. It is much more effective to provide them with a network, of volunteers, who will highlight other parts of their identity”.

## Conclusion

Based on in-depth interviews with high-profile ex-offenders and professionals working with them, the findings of this study illustrate the impact of the prison sentence and media attention on different life and social domains. Taken together, the nature of their crimes, the duration of their prison sentence, and the public attention to their cases post-release often inhibit these ex-offenders in building pro-social relationships post-release that may act as deterrents from future crime. From a life course theory perspective, this includes family relations, parenthood, intimate partner relationships, and employment. The limited presence of such pro-social roles urges life course theorist to turn to the broader (social) contexts of these ex-offenders for explaining desistance, and, as extension, adequate reintegration among this special group.

Even though this study attempted to reflect the lived experiences from a relatively small, hard-to-reach population, the recruitment strategy is not without limitations. First, we relied only on those who were willing to participate in our study. Those who—out of fear of renewed attention to their case or their person—did not participate may have reported different experiences. Second, we relied on self-reported crime alone, without the ability to cross-reference actual desistance with official reports. Even though we acknowledge that these ex-offenders may commit crimes for various reasons, we hold that self-reported crime is highly unlikely to go unnoticed among this group given their tight restrictions and constant monitoring and supervision [31]. Third, it should be noted that individual narratives constitute our unit of analysis and hence should be interpreted with caution. While constructing a narrative, these individuals may over-emphasize the role of external factors and downplay the influence of individual behavior. Future research should attempt to assess potential changes in narrative through longitudinal design—examining individual narratives at initial release from prison (in which all participants, including offenders without a high-profile), as well as after a follow-up of several years [31]. In this way, one could also assess to what extent (voluntary) employment, such as arranged for Johan, provides a “hook for change,” as suggested by life course theorists [14]. Further, while our study (partly because of the short time post-release) only focused on act-desistance, such a longitudinal design could allow for the assessment of other types of desistance including identity desistance and relational desistance.

Frequently being in the public eye, combined with strict supervision rules, seems to inhibit these individuals from rebuilding strong relationships that may act as informal social controls. While high-profile ex-offenders sometimes encounter different types of stigma, they frequently have comparable experiences on different life domains. First, from a life course theory point of view, establishing and maintaining an intimate relationship would be expected to positively influence these high-profile ex-offenders in going straight. The attachment to an intimate partner would give them a reluctance to engage in crime, because they might hurt or jeopardize their relationship [13]. Yet, similar to research conducted on paroled lifers (e.g., [28]), interviewees rarely indicated intimate partners as a positive influence, but rather pointed to stressors in the relationship because of public attention or the absence of intimate partners.

Second, from a life course perspective, (re)assuming a parental role is thought to positively influence these prisoner’s success post-release [13, 52]. Although the majority of the interviewees did not have any children, for those who did, either their children had become adults by the time they were released from prison or because the nature of their crime resulted in their (grand)children not wanting to be in contact.

A third factor derived from life course theory is the positive role of preexisting family ties on reentry. Similar to findings reported elsewhere, the prolonged, deprived nature of contact with family members while incarcerated may explain why so many families do not survive the imprisonment of one of their members ([1, 10]; Uggen et al. 2006). Further, reintegration into family life posed obstacles to these interviewees, as they were separated from family members for extended periods. To the extent that family members were alive upon their release, and willing to engage in contact, interviewees indicated they felt responsible for the stigmatizing impact their crimes had on their family members while they were incarcerated. Hence, rather being a source of support, existing family relations were absent or oftentimes constituted a source of concern. Further, to the extent that family members wished to be in contact, strict probation regulations and supervision oftentimes inhibited interviewees from engaging in family relations.

Fourth, mostly due to a combination of the nature of their crimes and the extended time spent behind bars, the vast majority of interviewees were not able to find paid employment.

In short, these ex-offenders’ desistance process seemed to occur irrespective of conventional life course factors, nor did post-release non-offending occur as a result of a change in male coming-of-age societal forces (e.g., parenthood, marriage, employment, and military service) [7], as emphasized by life course theorists [8, 13, 21, 32, 44, 50]. What seemed to occur, rather, was that these individuals were as Laub and Sampson described: “[making] a commitment to go straight without even realizing it.” These men, in short, they did not reengage in criminal behavior. In some cases, pro-social contacts (e.g., with volunteers) seem have played a role. At the same time, these contacts remained limited in scope. Rather than taking up new identities or (re)establishing relationships, the non-offending of many high-profile ex-offenders could also, at least in part, be attributed by other factors, including the prolonged period they spent behind bars and a coming-of-age of all men in the sample. Some scholars have pointed to biological and psychological factors such as bodily strength, physical energy, and psychological drive to explain the cessation of criminal behavior. One may question, however, to what extent these factors may explain the cessation in offending of these specific groups. Rather, age, in this regard, is a highly important factor as a proxy of changed lifestyles. In line with routine activities theory, older individuals are less likely to engage in crimes that have a relatively high risk of apprehension [49]—a risk that is exacerbated due to the intense, up-close supervision they experience.

Further, it may be argued that each of these men were sentenced and incarcerated for very specific crimes that took place in highly contextualized settings. Their long-term removal from these settings, in combination with both self-chosen as well as enforced social isolation, may explain their lack of reoffending.

In line with findings reported elsewhere [28, 38, 39, 53], our findings do not provide conclusive evidence that supervision prevents recidivism. In other words, we did not encounter any indication that public attention and strict supervision may have impacted directly and positively on the desistance process of these offenders.^2^ Equally, it cannot be demonstrated that post-release supervision was vital to the reintegration of these individuals. Rather, on the contrary, the majority of interviewees expressed that public attention indirectly, and post-release conditions directly forestalled their reentry, by having a negative influence on building and maintaining relationships and obtaining employment. These findings are in line with what Nugent and Schinkel [37] describe as “refrain[ing] from offending for long periods but not [being] in a position to establish a new identity” (p. 569). Ultimately, such excessive control can lead to a restricted and impoverished existence, previously described by Appleton [2] in her study on lifers on parole, who reported “a menial and lonely existence” (p. 117). Corresponding to findings reported elsewhere, the interviewed ex-prisoners’ identity was in a liminal state—they were no longer offenders, and no longer prisoners, but yet failed to achieve a new identity [37]. In our view, being in the public eye, combined with intense supervision, inhibited these men from establishing such a new identity through employment and relationships. This also accounted for volunteering, as for none of the interviewees this became an explicit part of their identity. This finding contrasts prior research describing the potential effect of volunteering and counseling in going straight [34]. In these studies, ex-offenders helping other ex-offenders considered themselves “super-counselors,” as because of their prison experience, they found themselves more capable than others to provide guidance and support, which in turn provided them meaning and a new identity [22, 26, 34], at times culminating into a new identity as a “professional ex”: those that make a living based on their prison experience, using their past in their daily lives to help other similarly stigmatized people. For ex-offenders in other studies [22, 26, 34], their past became a new part of their identity. Prior research suggests that this can even allow stigmatized individuals to overcome their felon label and reconcile with society for their past crimes [26]. Arguably, because of their high-profile status, reentry organizations offering volunteer positions may be reluctant in associating with participants in our sample and relying on their experiences, because of the nature of their offenses and associated high-profile status. This, in turn, may inhibit the creation of secondary (identity) desistance and tertiary (relational) desistance [36, 37].

In its current form, it has been argued strict supervision as experienced by these ex-offenders extends the gaze of the penal state and regulates and governs a group of marginalized people returning to their communities [28]. The vast majority of interviewees emphasized that they experienced the conditions as mechanisms of intense control, rather than as contributing to their rehabilitation. For many men, restrictions in housing and the experience of continuous surveillance led to isolation, which in turn inhibited them from establishing and maintaining social relationships, intimate relationships and employment. On a practical level, in short, supervision conditions often conflicted with the rebuilding their everyday lives.

Having trouble finding employment due to lack of professional expertise and protocol, experiencing difficulties in obtaining housing, and not being allowed to travel through certain areas contribute to what has recently been coined *the parole paradox* [40]. This suggests that while supervision aims to promote integration and desistance, it can actually place ex-offenders in a position that complicates efforts to attain a conventional lifestyle. It has been argued that repeated rejections by potential employers, repeated rejections by housing corporations and landlords, and restriction of movement by having to abide by supervision conditions can make these ex-offenders hyperaware of their status as ex-offender and hence inhibit their progress toward identity change [40]. In this way, such intense supervision accomplished the opposite of what it is supposed to do [28].

On a more fundamental level, truly making possible rehabilitation for this special group of offenders, one solution may lie in revising the current “contract” between the reentry organization and the (former) prisoner. Following Petersilia [38], based on her reflections of the US parole system, the focal point of such a revised contract must be a system of earned discharge, or accelerated release, whereby former prisoners have the ability to reduce the total length of their supervision by demonstrating arrest-free behavior and self-sufficiency. She argues that today’s post-release contract for ex-offenders spells out clearly the negative consequences that will be applied if they fail to comply with specified conditions. Rather, to accomplish and sustain behavioral change, research in various fields has shown the applicability of behavioral contracting [38]. A behavioral contract for such ex-offenders would be simply a written contract that specifies their behavioral obligations in meeting the terms of the contract and the supervisor’s obligations once the supervised ex-offenders have met theirs. Such a style of supervision—based on trust and dignity, within the framework of post-release requirements—gives the ex-offender the opportunity to strike out independently and to cast off their prison label and stigmatized identity.

Most ex-offenders, including the high-profile ex-offenders interviewed for this study, are motivated to be discharged from supervision. Research has demonstrated that older offenders, and individuals who stay arrest-free for 7 years or more, simply have very little risk for future crime, and this risk is similar to that of non-offenders [6]. Further, the existence of very long term supervision, combined with being in the eye of the public upon return, may create a hopeless environment that can trap an ex-offender and provide little incentive to adopt a pro-social attitude [6]. The revised supervision contract thus should combine both of these elements—behavioral change and accelerated supervision discharge—to produce tangible benefits for public safety and resource allocation and should be able to truly facilitate reentry of this group.

## Footnotes

1 Based on ethical considerations, especially the need for securing the identity of the respondents, the exact keywords are not mentioned in this article.

2 We thank one of the anonymous reviewers for pointing out this possibility
